# Foraging ants affect community composition and diversity of phyllosphere fungi on a myrmecophilous plants, *Mallotus japonicus*

**DOI:** 10.1002/ece3.11423

**Published:** 2024-05-15

**Authors:** Takafumi Mizuno, Hirotoshi Sato, Takao Itioka

**Affiliations:** ^1^ Graduate School of Human and Environmental Studies Kyoto University Kyoto Japan

**Keywords:** ants, fungal community, *Mallotus japonicus*, myrmecophilous plants, phyllosphere

## Abstract

Many microorganisms inhabit the aboveground parts of plants (i.e. the phyllosphere), which mainly comprise leaves. Understanding the structure of phyllosphere microbial communities and their drivers is important because they influence host plant fitness and ecosystem functions. Despite the high prevalence of ant–plant associations, few studies have used quantitative community data to investigate the effects of ants on phyllosphere microbial communities. In the present study, we investigated the effects of ants on the phyllosphere fungal communities of *Mallotus japonicus* using high‐throughput sequencing. *Mallotus japonicus* is a myrmecophilous plants that bears extrafloral nectaries, attracting several ant species, but does not provide specific ant species with nest sites like myrmecophytes do. We experimentally excluded ants with sticky resins from the target plants and collected leaf discs to extract fungal DNA. The ribosomal DNA internal transcribed spacer 1 (ITS1) regions of the phyllosphere fungi were amplified and sequenced to obtain fungal community data. Our results showed that the exclusion of ants changed the phyllosphere fungal community composition; however, the effect of ants on OTU richness was not clear. These results indicate that ants can change the community of phyllosphere fungi, even if the plant is not a myrmecophyte.

## Graphical abstract

We experimentally excluded ants with sticky resins from the target plants and collected leaf discs to extract fungal DNA. OTU based fungal community data showed that the exclusion of ants changed the phyllosphere fungal community composition; however, the effect of ants on OTU richness was not clear. These results indicate that ants can change the community of phyllosphere fungi, even if the plant is not a myrmecophyte.

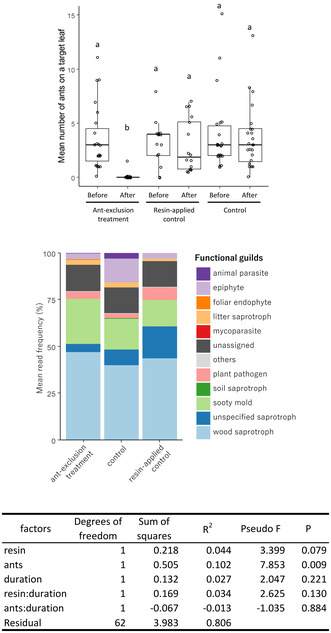

## 1 INTRODUCTION

The phyllosphere, surface and internal tissues of aboveground parts of plants, mainly comprise leaves populated by numerous microorganisms. Although the interactions between plants and microorganisms in the phyllosphere have been less studied than those in the rhizosphere, recent studies have shown that phyllosphere organisms exert significant effects on plant fitness and ecosystem functions (Bashir et al., 2022; Stone et al., 2018; Vacher et al., 2016). In addition to the importance of phyllosphere microorganisms in plant survival, growth, and reproduction, the phyllosphere is considered an ideal model system for testing ecological concepts (Meyer & Leveau, 2012). Therefore, many recent studies have investigated the structure and drivers of phyllosphere microbial communities.

Fungi are the primary components of the phyllosphere microbiome (Bashir et al., 2022; Stone et al., 2018; Vacher et al., 2016). Some phyllosphere fungi act as plant pathogens (Doehlemann et al., 2017), whereas others provide host plants with tolerance to several types of stress such as desiccation, UV exposure, and pathogens (Bashir et al., 2022; Stone et al., 2018; Vacher et al., 2016). Phyllosphere fungi can affect the fitness of their host plants and other fungi also play a role in leaf decomposition during the early stage (Osono, 2006; Unterseher et al., 2013; Voříšková & Baldrian, 2013). Many studies have explored this effect of phyllosphere fungi on plants and ecosystem functioning, and results have shown that the compositions of phyllosphere fungal communities are influenced by many factors. Leaf morphology, chemistry, and physiology differ among plant genotypes and species. Many studies have shown phyllosphere fungal communities varies among plant genotypes (Bálint et al., 2013; Cordier, Robin, Capdevielle, Desprez‐Loustau, & Vacher, 2012; Horton et al., 2014; Qian et al., 2018; Sapkota et al., 2015) and plant species (Kembel & Mueller, 2014; Qian et al., 2020; Sapkota et al., 2015; Yao et al., 2019). The leaf microclimate varies with regional climate and leaf position within a plant due to landscape, vegetation, and canopy structure. A significant effect of leaf microclimate on phyllosphere fungal communities has also shown (Bálint et al., 2015; Coince et al., 2014; Cordier, Robin, Capdevielle, Desprez‐Loustau, & Vacher, 2012; Cordier, Robin, Capdevielle, Fabreguettes, et al., 2012; Osono, 2014). In addition to these abiotic factors, the effect of interactions among microorganisms on phyllosphere fungi has been inferred by studies mostly in the context of disease biocontrol (Becker et al., 2020; Braun‐Kiewnick et al., 2000; Innerebner et al., 2011; Prior et al., 2017). Moreover, insects that interact with plants can affect phyllosphere fungal communities.

Many plants of a wide range of lineages are associated with ants that protect them against herbivores. Some plants, known as myrmecophytes, provide specific ant partners with nesting sites (domatia). Most other plants, known as myrmecophilous plants, lack domatia but associate with various ant species through extrafloral nectaries (EFNs), which secrete nutrient liquids for ants (Marazzi et al., 2013). Because ants mechanically remove microbes and secrete antibiotic substances from several glands (Offenberg & Damgaard, 2019), the effects of ants on plant pathogens have been suggested to be potentially high (Offenberg & Damgaard, 2019). Several studies have shown that ants regulate plant pathogens; most of these studies use myrmecophytes (Belin‐Depoux et al., 1997; Heil et al., 1999, 2001; Letourneau, 1998; Roux et al., 2011; Thornham et al., 2012). However, studies that investigate the effect of ants on the fungal phyllosphere community are lacking and very few studies used myrmecophilous plants to investigate the effect of ants attracted to EFNs on pathogens (de la Fuente & Marquis, 1999) despite the prevalence of non‐obligate associations between ants and myrmecophilous plants.

In this study, we investigated whether ants influence the species richness and community composition of phyllosphere fungi using high‐throughput sequencing. The focal plant species was the East Asian deciduous shrub *Mallotus japonicus* (Thunb.) Muell. Arg. (Euphorbiaceae). *Mallotus japonicus* is a myrmecophilous plants that bears EFNs on its leaves for indirect defense by ants (Yamawo et al., 2014; Yamawo, Katayama, et al., 2012; Yamawo, Suzuki, et al., 2012). To assess the effect of ants on the phyllosphere fungal communities we performed experimental manipulations to control the presence of ants on the leaves. Here, we tested the three hypotheses, (1) ants decrease operational taxonomic unit (OTU) richness of phyllosphere fungi, (2) ants affect phyllosphere fungal community composition, and (3) ants affect different functional guilds of fungi differently, specifically decreasing plant pathogens.

## 2 MATERIALS AND METHODS

### 2.1 Study site and sample collection

Samples were collected in Mt. Yoshida (35°01′31″ N, 135°47′09″ E), Kyoto, and Japan. We selected three open sites as the study plots, all of which fell within a radius of 500 m but were at least 50 m apart. We selected 69 saplings of *M. japonicus* (50–150 cm in height), of which 35, 18, and 16 were located in each of the three plots. The youngest, fully expanded leaf was selected as the target leaf for each sapling because leaves of this age attract more ants than those of other ages (Yamawo, Suzuki, et al., 2012).

Next, we randomly assigned 27, 16, and 26 of the total 69 saplings to the “ant‐exclusion treatment”, “resin‐applied control”, and “control” groups, respectively (see Appendix S1 for the details). In the ant‐exclusion treatment, we applied sticky resin (Fuji Tangle; Fuji Yakuhin Co., Ltd., Saitama, Japan) to the stems and petioles of each target sapling and cut the leaves and stems of surrounding plants to prevent ants from accessing each target leaf. In the resin‐applied control, we applied sticky resin to each target sapling and then placed a small twig onto the applied resin or attached a part of the surrounding plant to each sapling to allow ants to access the target leaf on each sapling through the twig or the plant. For the control treatment, we maintained each target sapling without any manipulation. These experimental manipulations started between June 27 and July 16, 2021 and ended in 2 or 4 weeks (see below), and enabled us to distinguish the effects of ant exclusion from those of resin application. Before the experiment started, we observed the target leaves on all saplings, counted the number of ants and identified them on each target leaf. After the experiment started, we repeated this observation one to four times. The variation in observation frequency among the saplings was due to weather conditions; observations were suspended whenever it rained, as rain reduces ant activity. This was done to check whether each manipulation controlled ant presence on the leaf as intended.

Two or 4 weeks after sapling manipulation, between July 11 and August 6, 2021, we sampled leaf discs to assess both endophytic and epiphytic fungi. All saplings were allocated to either a two‐week or a four‐week experiment (see Appendix S1), indicating that the leaf discs were collected just once from each sapling. For fungal sampling, we clipped a leaf disc approximately 1.0 cm in diameter from an intact part of the leaf area at approximately 2 cm from the leaf base of each leaf using a hole puncher and forceps and then placed the leaf disc into a sterile 2 mL microtube using forceps. The hole puncher and forceps were carefully wiped with alcohol wipes after manipulating a leaf disc and before collecting another leaf disc.

### 2.2 DNA extraction, polymerase chain reaction (PCR) amplification, and DNA sequencing

Total DNA from both endophytic and epiphytic fungi was extracted from the leaf discs using the cetyltrimethylammonium bromide (CTAB) method. Each leaf disc was crushed in a 2.0 mL microtube using a TissueLyser II (QIAGEN) and a zirconia bead of 5 mm diameter. Then, 500 μL of CTAB buffer (10 mg CTAB; 100 mM Tris; 41 mg NaCl; 20 mM EDTA) was added to the tubes and the mixture was incubated for 30 min at 55°C. After incubation, the suspension was mixed with 500 μL of CIA solution (chloroform: isoamyl alcohol = 24:1) and centrifuged for 5 min at 20°C and 20,630 × *g*. We transferred 300 μL of the supernatant to a new microtube. The supernatant and 300 μL of ice‐cold isopropanol were mixed and centrifuged for 10 min at 4°C and 20,630  × *g*. After being washed with 70% ethanol, the precipitated DNA was dissolved in 200 μL of TE buffer (10 mM Tris; 1 mM EDTA, pH 8.0).

The internal transcribed spacer 1 (ITS1) regions of ribosomal DNA were amplified using the primer pairs ITS1F_KYO2 and ITS2_KYO2 (Toju et al., 2012) fused with an Illumina sequencing primer and six random bases (N). Polymerase chain reaction was performed using KOD FX Neo (TOYOBO, Osaka, Japan). The reaction solution of 10 μL contained 1 × PCR buffer, 0.4 mM deoxynucleoside triphosphates, 0.3 μM each of the forward and reverse primers, and 0.2 units of KOD FX Neo polymerase. The PCR conditions were as follows: an initial denaturation for 2 min at 94°C; followed by 40 cycles of 10 s at 98°C, 30 s at 60°C, 30 s at 68°C; and a final extension for 5 min at 68°C. To subsequently fuse the 8 bp identifier indices (Hamady et al., 2008) and the MiSeqP5/P7 adapter to the initial PCR amplicons, we conducted an additional PCR using the same PCR mixture and conditions as the initial PCR, except that the number of cycles was reduced to 12. The resulting PCR amplicons were pooled and purified using AMPure XP (Beckman Coulter, Brea, CA, USA). The purified library was excised using E‐Gel SizeSelect (Thermo Fisher Scientific, Waltham, MA, USA). The libraries were sequenced via 2 × 250‐bp paired‐end sequencing on a MiSeq platform (Illumina, San Diego, CA, USA) using the MiSeq Reagent Kit v2 (500 cycles), following the manufacturer's instructions.

### 2.3 Bioinformatics

The reads retrieved from MiSeq sequencing were processed using CLAIDENT version 0.2.2019.04.27 (Tanabe & Toju, 2013). Reads were de‐multiplexed using the “clsplitseq” command and the resulting reads were deposited in the Sequence Read Archive of the DNA Data Bank of Japan (accession number: DRA017572). Using the “clfilterseq” command, low‐quality reads were filtered based on a minimum quality value of 30. The resulting forward and reverse reads were merged using the “clconcatpair” command. Noisy sequences were eliminated using the “clcleanseqv” command. The remaining reads were clustered into operational taxonomic units (OTUs) with similarity thresholds of 0.97 using the “clclassseqv” command. Potential chimeric OTUs were eliminated using UCHIME (Edgar et al., 2011) without any references. Each OTU was identified through a local BLAST search using the “clidentseq” command and the “classigntax” command in CLAIDENT.

### 2.4 Data analysis

All statistical analyses were conducted using R version 4.3.1 (R Core Team, 2023). To test the effect of the experimental manipulations on ant abundance on the target leaves, we compared the number of ants between six groups, which were combinations of the three treatments (ant‐exclusion treatment, resin‐applied control, and control) and two periods (before and after manipulation) using the Steel–Dwass test. The mean number of ants for each target leaf was used as a response variable when observations were conducted more than once. We also assessed if species richness and species composition of the ants were different between before and after the manipulation in resin‐applied control (see Appendix S2 for the details).

To determine the overall fungal composition, OTU richness, and relative read abundance were calculated. For OTU relative read abundance, the OTU read abundance in each sample was converted into a percentage, and then, the mean of percentages for an OTU was calculated to correct for the difference in the total OTU read abundance between leaf disc samples.

To analyze the phyllosphere fungal community, we first removed data of samples that had less than 1000 total reads after sequence data processing. Thereafter, the reads were resampled based on the smallest coverage among the remaining samples using the *rarefy* function. A distance matrix of the fungal community was constructed by resampling the data using Raup–Crick dissimilarities. We conducted a permutational multivariate analysis of variance (PERMANOVA) (Anderson, 2001) with three factors, “ants” (two levels: ants present and absent), “resin” (two levels: applied and not applied), and “experimental duration” (two levels: 2 and 4 weeks) and two interaction terms, “ants” × “experimental duration” and “resin” × “experimental duration.” We set the plot as a block so that sample units were permuted only within a block and ran PERMANOVA with 9999 permutations with the *adonis2* function. All functions used for the fungal community analyses described above were performed using the package *vegan* in R (Oksanen et al., 2020).

We also calculated the asymptotic estimates of OTU richness for each sample using the *iNEXT* function (Chao et al., 2014; Hsieh et al., 2016). To test the effect of treatments and experimental duration on the estimated fungal OTU richness, *ANOVA* using a linear mixed model with the *lmer* function in the package *lmerTest* (Kuznetsova et al., 2017) was performed. For the linear mixed model, “treatment” (three levels: ant‐exclusion treatment, resin‐applied control, and control), “experimental duration” (two levels: 2 and 4 weeks), and an interaction term of these were set as explanatory variables and the plot was set as a random factor. To contrast the differences between the combinations of treatments and the experimental duration, the *emmeans* function in the package *emmeans* in R was used (Lenth, 2023).

To assess if ants affect frequency of specific fungal guilds, *FungalTraits* (Põlme et al., 2020) was used to associate potential functions to the fungal OTUs based on their taxonomy at the level of genera. Analyses of guild‐based community composition were not performed, instead a simpler and more conservative analysis, Kruskal–Wallis test, was performed due to the high rate of unassigned guild OTUs, which accounted for more than 10% of all reads on average. We excluded eight samples (three from control, three from ant‐exclusion treatment and two from resin‐applied control) because over 30% of the reads could not be assigned to any guild. Subsequently we performed Kruskal–Wallis test to evaluate the difference in the rate of each guild between the three experimental treatments, ant‐exclusion treatment, resin‐applied control, and control. The Kruskal–Wallis test was performed for five abundant fungal guilds, epiphyte, plant pathogen, sooty mold, unspecified saprotroph, and wood saprotroph.

## 3 RESULTS

**FIGURE 1:**
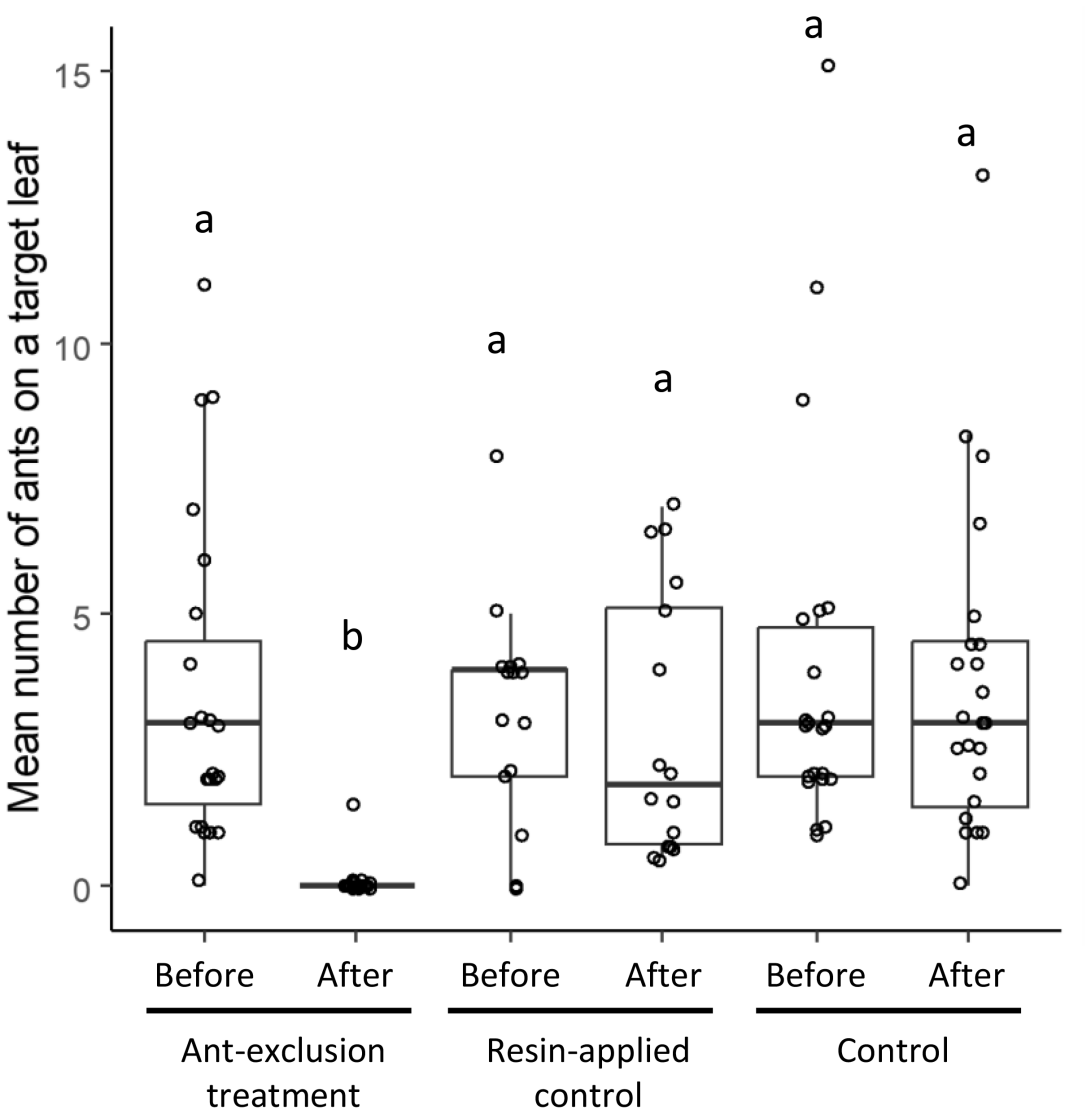
Comparison of the number of ants on target leaves between the treatments before and after the beginning of the experimental manipulations. Different letters above each boxplot indicate a significant difference (*p* < .001) by the Steel‐Dwass test.

We identified 21 ant species on the target leaves (Appendix S3). The experimental manipulation controlled the presence of ants on the target leaves as intended (Figure 1). The number of ants on the target leaves significantly decreased and became zero in all cases except one after the application of sticky resin in the ant‐exclusion treatment (*p* < .001), indicating that the applied resin prevented ants from accessing the leaves. However, the number of ants in the resin‐applied control was not significantly different before and after manipulation, indicating that ants were still able to access the leaves after the application of the resin. We also confirmed that species richness and composition of the ants in resin‐applied control were not significantly different between before and after manipulation (Appendix S2).

A total of 872 fungal OTUs were detected in all the samples. The total OTU richness of Ascomycota was higher than that of Basidiomycota; however, the relative OTU read abundance was higher in Basidiomycota than in Ascomycota (Table 1). At the genus level, more than 70% of the relative OTU read abundances consisted of the five most abundant genera *Curvibasidium*, *Aureobasidium*, *Cryptococcus*, *Aotearoamyces*, and *Pseudozyma*, which included only 17 OTUs (Table 1).

**TABLE 1:** Operational taxonomic unit (OTU) richness and OTU read abundance at the plylum and genus levels in the *Mallotus japonicus* phyllosphere.

Taxonomic level	Taxon	OTU richness	OTU read abundance (%)
Phylum	Basidiomycota	345	61.1
	Ascomycota	522	38.6
	Unknown	5	0.36
Genus	Curvibasidium	4	35.4
	Aureobasidium	6	18.2
	Cryptococcus	4	8.48
	Aotearoamyces	1	7.24
	Pseudozyma	2	4.51
	Botryosphaeria	1	1.97
	Cladosporium	3	1.95
	Filobasidium	5	1.52
	Others	494	6.5
	Unknown	352	14.2

**FIGURE 2:**
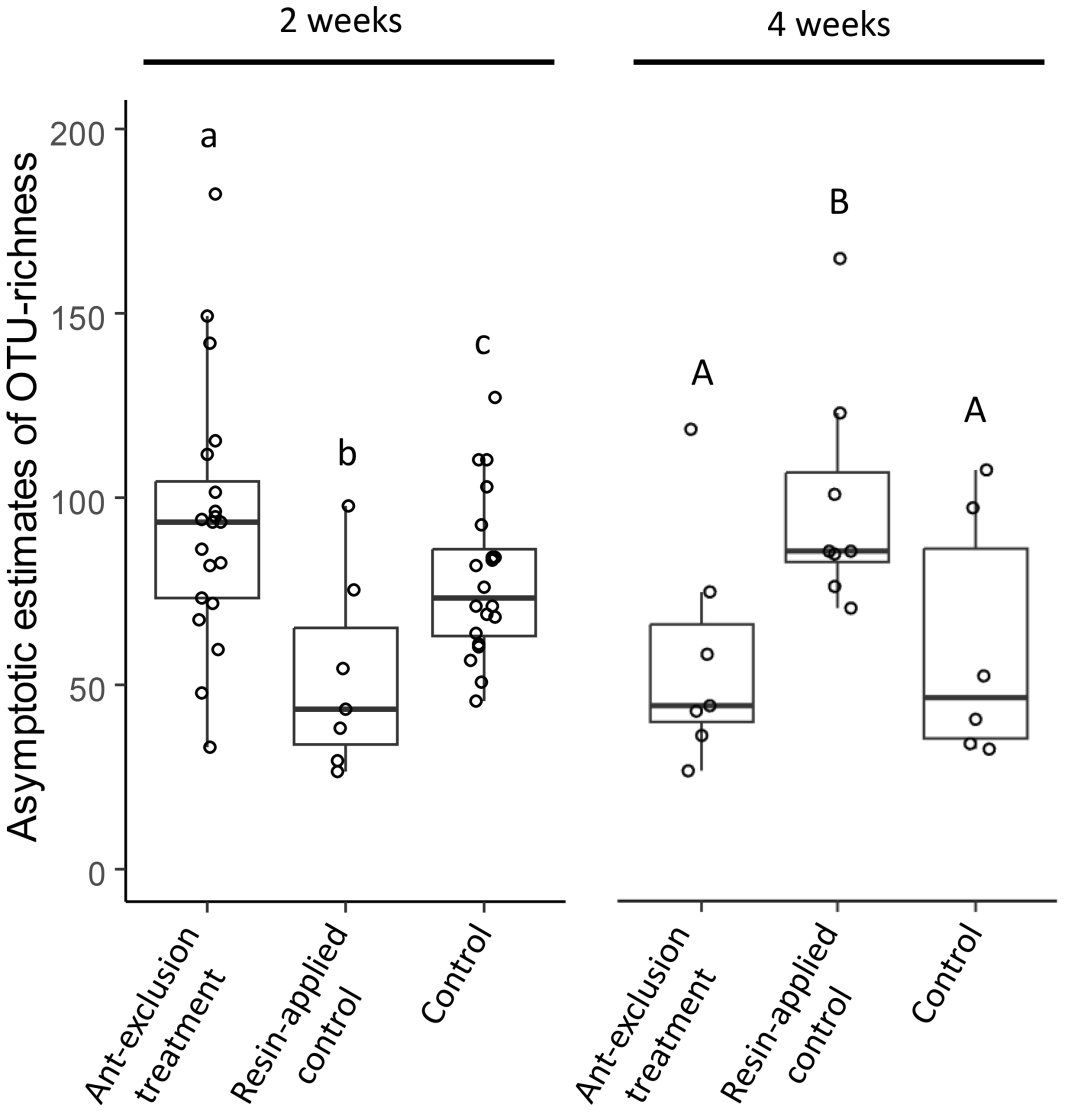
Estimated operational taxonomic unit (OTU) richness of the three treatments for the two experimental durations. Different letters above each boxplot indicate a significant difference (*p* < .05) within each experimental duration.

Operational taxonomic unit richness in the fungal communities was not significantly affected by treatment or experimental duration (*F* = 1.05, *p* = .36, and *F* = 1.69, *p* = .20, respectively); however, the interaction between them had a significant effect (*F* = 26.5, *p* < .001). The significant interaction effect corresponded to the finding that the patterns of differences in OTU richness among the three treatments differed between the two durations. In the 2‐week duration experiment, OTU richness in the ant‐exclusion treatment and resin‐applied controls was significantly higher and lower, respectively, than that in the control (Figure 2). However, in the 4‐week duration experiment, OTU richness in the ant‐exclusion treatment was not significantly different from that in the control, and in the resin‐applied control OTU richness was significantly higher than that in the control (Figure 2).

Results of PERMANOVA showed that ants significantly affected the phyllosphere fungal community composition, although the coefficient of determination was not very large (*R* ^2^ = .102), whereas the coefficient of determination of residuals was large (*R* ^2^ = .806, Table 2). None of the other explanatory variables had significant effects (Table 2).

**TABLE 2:** Summary of PERMANOVA analysis result for the phyllosphere fungal community.

Factors	Degrees of freedom	Sum of squares	*R* ^2^	Pseudo F	*p*
Resin	1	0.218	.044	3.399	.079
Ants	1	0.505	.102	7.853	.009
Duration	1	0.132	.027	2.047	.221
Resin: duration	1	0.169	.034	2.625	.130
Ants: duration	1	−0.067	−.013	−1.035	.884
Residual	62	3.983	.806		

**FIGURE 3:**
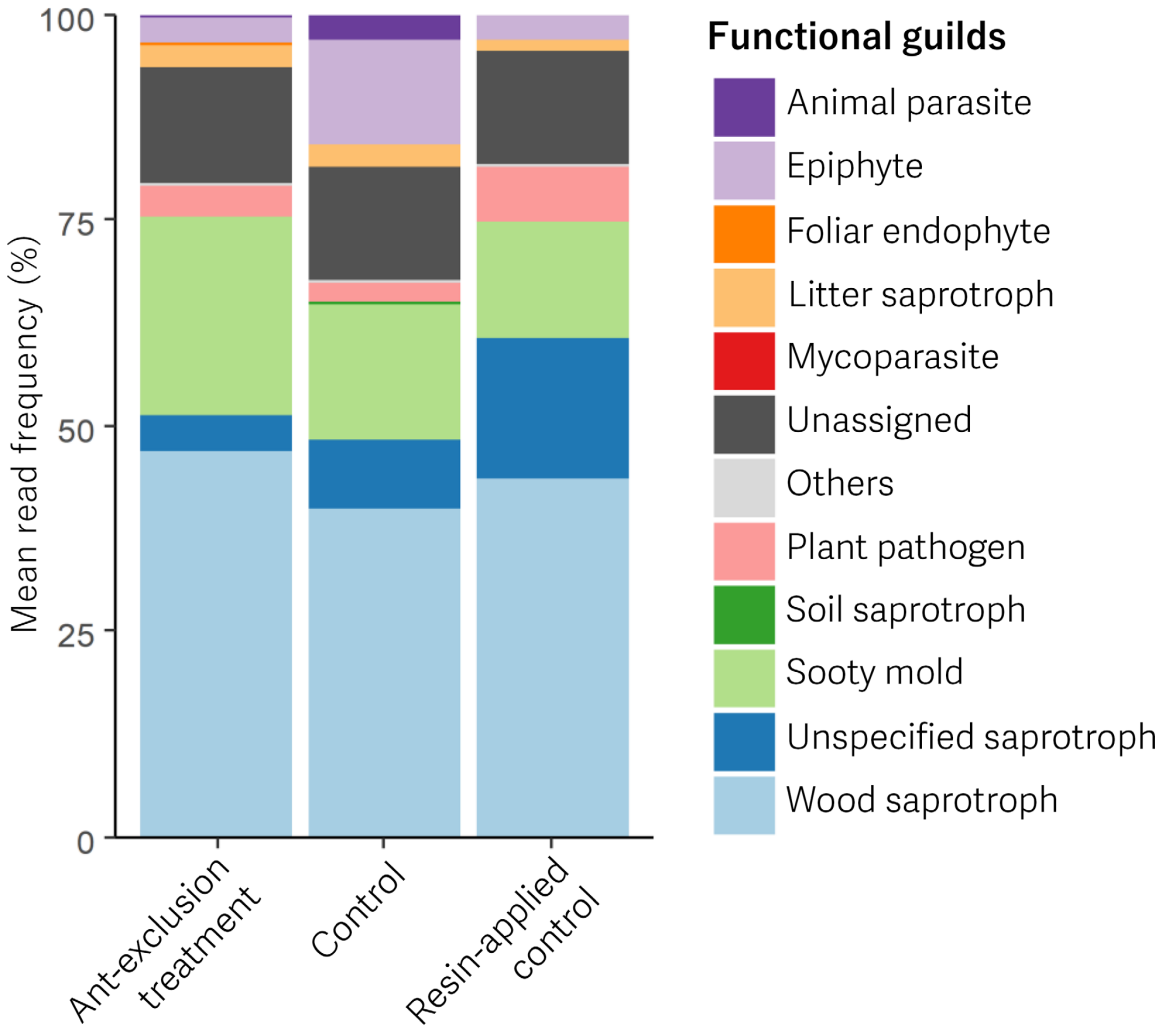
Mean read frequency of each functional guilds in the three experimental treatments.

Most abundant fungal guild was wood saprotroph in all treatments (Figure 3). The four guilds, sooty mold, unspecified saprotroph, epiphyte and plant pathogen, were also abundant (Figure 3). We detected no significant difference in rate of the guilds between the treatments for epiphyte (χ^2^ = 4.05, *p* = .13), plant pathogen (χ^2^ = 2.72, *p* = .18), sooty mold (χ^2^ = 3.39, *p* = .18), unspecified saprotroph (χ^2^ = 0.48, *p* = .79) and wood saprotroph (χ^2^ = 0.20, *p* = .91).

## 4 DISCUSSION

As hypothesized, our results showed that the phyllosphere fungal community composition of *M. japonicus* was significantly affected by the presence of ants attracted to EFNs on the leaves. Antibiotics and the cleaning removal behavior of the ants, which have been reported as mechanisms that affect microorganisms (Offenberg & Damgaard, 2019), are likely to play a significant role in shaping fungal community composition. To the best of our knowledge, this is the first study to show the effect of ants on the phyllosphere fungal community composition based on robust quantitative evidence using OTU‐based community data. Many previous studies have shown that ants reduce certain phyllosphere fungi, for example *Fusarium verticillioides* (formerly *Fusarium moniliforme*) (Belin‐Depoux et al., 1997; Roux et al., 2011), *Pestalotia* sp. (de la Fuente & Marquis, 1999) and *Elsinoe mangiferae* (Peng & Christian, 2005), but they have not assessed the entire community of phyllosphere fungi. Most of these studies focused on a small number of specific plant pathogens and assessed the reduction in pathogens based on visible evidence, such as fungal infestation and host plant symptoms caused by the pathogens (de la Fuente & Marquis, 1999; Heil et al., 1999, 2001; Letourneau, 1998; Peng & Christian, 2005, 2013; Roux et al., 2011; Thornham et al., 2012). Another study showed a reduction in the abundance of phyllosphere fungi by cultivation but did not assess community composition (González‐Teuber & Heil, 2010). Only one study used OTU‐based community data and showed that phyllosphere bacterial community composition was affected by ants (González‐Teuber et al., 2014).

Contrary to our hypothesis, the rates of the five abundant functional guilds including plant pathogens and sooty mold were not significantly affected by the experimental treatments. Considering many studies that have shown that ants reduce specific plant pathogens, *Fusarium verticillioides* (formerly *Fusarium moniliforme*) (Belin‐Depoux et al., 1997; Roux et al., 2011), *Pestalotia* sp. (de la Fuente & Marquis, 1999), *Elsinoe mangiferae* (Peng & Christian, 2005) and unidentified plant pathogens (González‐Teuber et al., 2014; González‐Teuber & Heil, 2010; Heil et al., 1999, 2001; Letourneau, 1998; Peng & Christian, 2013; Thornham et al., 2012), ants probably affect each fungal species differently even within the same functional guild.

For OTU richness, the main effect of the two explanatory variables, treatment, and experimental duration, was unclear because the tendency in OTU richness between the treatments differed according to the experimental duration; this difference seems to obscure the effect of ants on fungal OTU richness. The OTU richness in the ant‐exclusion treatment was expected to be higher than that in the control, considering the plausible effects of ant antibiotics (Offenberg & Damgaard, 2019) on the fungi. This hypothesis is likely correct for a 2‐week duration, but not for a 4‐week duration. Fungi that can be excluded from the phylospheric environment by ant activity may inhibit other fungi from colonizing ant‐excluded leaves through interspecific competition and may have caused a reduction in OTU richness within 4 weeks after ant exclusion. The elusive behavior of OTU richness in the resin‐applied treatment was difficult to interpret, but it was possibly caused by both stochastic factors and the relatively small sample size.

It is noteworthy that our target plant, *M. japonicus*, is not a myrmecophytic species, and there were no ant nests on the target plant saplings, which suggests that fungal community composition can be changed by ants even when the plant is not a myrmecophyte and not inhabited by ant colonies. To date, only one study has showed that ants visiting plants from their nests regulate leaf pathogen damage (de la Fuente & Marquis, 1999). On the other hand, almost all studies of the effect of ants on pathogen or phyllosphere microbiomes used myrmecophytes or plants with arboreal ant nests (Belin‐Depoux et al., 1997; González‐Teuber et al., 2014; González‐Teuber & Heil, 2010; Heil et al., 1999, 2001; Letourneau, 1998; Peng & Christian, 2005, 2013; Roux et al., 2011; Thornham et al., 2012).

However, despite such studies about myrmecophytes, few study assessed entire phyllosphere fungal communities of myrmecophytes. Lucas et al. (2019) demonstrated that bacterial and fungal community compositions varied across different parts of the myrmecophyte species *Cecropia peltata*, highlighting the ability of ants on the phyllosphere microbial communities. However, the exact impact of ant presence remains unclear, as ant‐exclusion experiments were not conducted. Considering the prevalence of ant‐plant interactions, the available data of ant effect on phyllosphere microbial community in myrmecophytes and myrmecophilous plants are still very limited. Assessing the difference of the ant effect between myrmecophilous plants and myrmecophytes with more data would be intriguing and could advance our understanding of the ecology and evolution of ant‐plant relationships.

## AUTHOR CONTRIBUTIONS

**Takafumi Mizuno:** Conceptualization (equal); data curation (equal); formal analysis (equal); investigation (equal); methodology (equal); project administration (equal); visualization (equal); writing – original draft (equal). **Hirotoshi Sato:** Conceptualization (equal); data curation (equal); writing – review and editing (equal). **Takao Itioka:** Conceptualization (equal); funding acquisition (lead); project administration (lead); writing – review and editing (equal).

## CONFLICT OF INTEREST STATEMENT

All authors have no conflict of interest.

## Supporting information


Appendix S1.

Appendix S2.

Appendix S3.

## Data Availability

The data that support the findings of this study are openly available in DDBJ Sequence Read Archive (DRA) at https://www.ddbj.nig.ac.jp/dra/index‐e.html, reference number DRA017572.

## References

[ece311423-bib-0001] Anderson, M. J. (2001). A new method for non‐parametric multivariate analysis of variance. Austral Ecology, 26(1), 32–46.

[ece311423-bib-0002] Bálint, M. , Bartha, L. , O'Hara, R. B. , Olson, M. S. , Otte, J. , Pfenninger, M. , Robertson, A. L. , Tiffin, P. , & Schmitt, I. (2015). Relocation, high‐latitude warming and host genetic identity shape the foliar fungal microbiome of poplars. Molecular Ecology, 24(1), 235–248. 25443313 10.1111/mec.13018

[ece311423-bib-0003] Bálint, M. , Tiffin, P. , Hallström, B. , O'Hara, R. B. , Olson, M. S. , Fankhauser, J. D. , Piepenbring, M. , & Schmitt, I. (2013). Host genotype shapes the foliar fungal microbiome of balsam poplar (*Populus balsamifera*). PLoS One, 8(1), e53987. 23326555 10.1371/journal.pone.0053987 PMC3543377

[ece311423-bib-0004] Bashir, I. , War, A. F. , Rafiq, R. Z. A. , Rashid, I. , & Shouche, Y. S. (2022). Phyllosphere microbiome: Diversity and functions. Microbiological Research, 254, 126888. 34700185 10.1016/j.micres.2021.126888

[ece311423-bib-0005] Becker, R. , Ulrich, K. , Behrendt, U. , Kube, M. , & Ulrich, A. (2020). Analyzing ash leaf‐colonizing fungal communities for their biological control of *Hymenoscyphus fraxineus* . Frontiers in Microbiology, 11, 590944. 33193255 10.3389/fmicb.2020.590944 PMC7649789

[ece311423-bib-0006] Belin‐Depoux, M. , Solano, P. J. , Lubrano, C. , Robin, J. R. , Chouteau, P. , & Touzet, M. C. (1997). La fonction myrmécophile de *Cecropia obtusa* Trecul (*Cecropiaceae*) en Guyane française. Acta Botanica Gallica, 144(3), 289–313.

[ece311423-bib-0007] Braun‐Kiewnick, A. , Jacobsen, B. J. , & Sands, D. C. (2000). Biological control of *pseudomonas syringae* pv. Syringae, the causal agent of basal kernel blight of barley, by antagonistic *Pantoea agglomerans* . Phytopathology, 90(4), 368–375. 18944586 10.1094/PHYTO.2000.90.4.368

[ece311423-bib-0008] Chao, A. , Gotelli, N. J. , Hsieh, T. C. , Sander, E. L. , Ma, K. H. , Colwell, R. K. , & Ellison, A. M. (2014). Rarefaction and extrapolation with hill numbers: A framework for sampling and estimation in species diversity studies. Ecological Monographs, 84(1), 45–67.

[ece311423-bib-0009] Coince, A. , et al. (2014). Leaf and root‐associated fungal assemblages do not follow similar elevational diversity patterns. PLoS One, 9(6), e100668. 24971637 10.1371/journal.pone.0100668 PMC4074112

[ece311423-bib-0010] Cordier, T. , Robin, C. , Capdevielle, X. , Desprez‐Loustau, M.‐L. , & Vacher, C. (2012). Spatial variability of phyllosphere fungal assemblages: Genetic distance predominates over geographic distance in a European beech stand (*Fagus sylvatica*). Fungal Ecology, 5(5), 509–520.

[ece311423-bib-0011] Cordier, T. , Robin, C. , Capdevielle, X. , Fabreguettes, O. , Desprez‐Loustau, M. L. , & Vacher, C. (2012). The composition of phyllosphere fungal assemblages of European beech (*Fagus sylvatica*) varies significantly along an elevation gradient. New Phytologist, 196(2), 510–519. 22934891 10.1111/j.1469-8137.2012.04284.x

[ece311423-bib-0012] de la Fuente, M. A. S. , & Marquis, R. J. (1999). The role of ant‐tended extrafloral nectaries in the protection and benefit of a neotropical rainforest tree. Oecologia, 118, 192–202. 28307694 10.1007/s004420050718

[ece311423-bib-0013] Doehlemann, G. , Ökmen, B. , Zhu, W. , & Sharon, A. (2017). Plant pathogenic fungi. Microbiology Spectrum, 5(1), 5114. 10.1128/microbiolspec.funk-0023-2016 PMC11687436 28155813

[ece311423-bib-0014] Edgar, R. C. , Haas, B. J. , Clemente, J. C. , Quince, C. , & Knight, R. (2011). UCHIME improves sensitivity and speed of chimera detection. Bioinformatics, 27(16), 2194–2200. 21700674 10.1093/bioinformatics/btr381 PMC3150044

[ece311423-bib-0015] González‐Teuber, M. , & Heil, M. (2010). Pseudomyrmex ants and acacia host plants join efforts to protect their mutualism from microbial threats. Plant Signaling & Behavior, 5(7), 890–892. 20484982 10.4161/psb.5.7.12038 PMC3014543

[ece311423-bib-0016] González‐Teuber, M. , Kaltenpoth, M. , & Boland, W. (2014). Mutualistic ants as an indirect defence against leaf pathogens. New Phytologist, 202(2), 640–650. 24392817 10.1111/nph.12664

[ece311423-bib-0017] Hamady, M. , Walker, J. J. , Harris, J. K. , Gold, N. J. , & Knight, R. (2008). Error‐correcting barcoded primers for pyrosequencing hundreds of samples in multiplex. Nature Methods, 5(3), 235–237. 18264105 10.1038/nmeth.1184 PMC3439997

[ece311423-bib-0018] Heil, M. , Fiala, B. , Linsenmair, K. E. , & Boller, T. (1999). Reduced chitinase activities in ant plants of the genus macaranga. Naturwissenschaften, 86(3), 146–149.

[ece311423-bib-0019] Heil, M. , Fiala, B. , Maschwitz, U. , & Linsenmair, K. E. (2001). On benefits of indirect defence: Short‐and long‐term studies of antiherbivore protection via mutualistic ants. Oecologia, 126, 395–403. 28547454 10.1007/s004420000532

[ece311423-bib-0020] Horton, M. W. , Bodenhausen, N. , Beilsmith, K. , Meng, D. , Muegge, B. D. , Subramanian, S. , Vetter, M. M. , Vilhjálmsson, B. J. , Nordborg, M. , Gordon, J. I. , & Bergelson, J. (2014). Genome‐wide association study of *Arabidopsis thaliana* leaf microbial community. Nature Communications, 5(1), 5320. 10.1038/ncomms6320 PMC4232226 25382143

[ece311423-bib-0021] Hsieh, T. C. , Ma, K. H. , & Chao, A. (2016). iNEXT: An R package for rarefaction and extrapolation of species diversity (hill numbers). Methods in Ecology and Evolution, 7(12), 1451–1456.

[ece311423-bib-0022] Innerebner, G. , Knief, C. , & Vorholt, J. A. (2011). Protection of *Arabidopsis thaliana* against leaf‐pathogenic *Pseudomonas syringae* by Sphingomonas strains in a controlled model system. Applied and Environmental Microbiology, 77(10), 3202–3210. 21421777 10.1128/AEM.00133-11 PMC3126462

[ece311423-bib-0023] Kembel, S. W. , & Mueller, R. C. (2014). Plant traits and taxonomy drive host associations in tropical phyllosphere fungal communities. Botany, 92(4), 303–311.

[ece311423-bib-0024] Kuznetsova, A. , Brockhoff, P. B. , & Christensen, R. H. B. (2017). lmerTest package: Tests in linear mixed effects models. Journal of Statistical Software, 82(13), 1–26.

[ece311423-bib-0025] Lenth, R. V. (2023). Emmeans: Estimated marginal means, aka least‐squares means.

[ece311423-bib-0026] Letourneau, D. K. (1998). Ants, stem‐borers, and fungal pathogens: Experimental tests of a fitness advantage in piper ant‐plants. Ecology, 79(2), 593–603.

[ece311423-bib-0027] Lucas, J. M. , Madden, A. A. , Penick, C. A. , Epps, M. J. , Marting, P. R. , Stevens, J. L. , Fergus, D. J. , Dunn, R. R. , & Meineke, E. K. (2019). Azteca ants maintain unique microbiomes across functionally distinct nest chambers. Proceedings of the Royal Society B: Biological Sciences, 286, 20191026. 10.1098/rspb.2019.1026 PMC6710589 31387509

[ece311423-bib-0028] Marazzi, B. , Bronstein, J. L. , & Koptur, S. (2013). The diversity, ecology and evolution of extrafloral nectaries: Current perspectives and future challenges. Annals of Botany, 111(6), 1243–1250. 23704115 10.1093/aob/mct109 PMC3662527

[ece311423-bib-0029] Meyer, K. M. , & Leveau, J. H. J. (2012). Microbiology of the phyllosphere: A playground for testing ecological concepts. Oecologia, 168(3), 621–629. 21983641 10.1007/s00442-011-2138-2 PMC3277708

[ece311423-bib-0030] Offenberg, J. , & Damgaard, C. (2019). Ants suppressing plant pathogens: A review. Oikos, 128(12), 1691–1703.

[ece311423-bib-0031] Oksanen, J. , Blanchet, G. , Friendly, M. , Kindt, R. , Legendre, P. , McGlinn, D. , Minchin, P. , O'Hara, R. , Simpson, G. , & Solymos, P. (2020). Vegan: Community ecology package 2.5‐7. R package.

[ece311423-bib-0032] Osono, T. (2006). Role of phyllosphere fungi of forest trees in the development of decomposer fungal communities and decomposition processes of leaf litter. Canadian Journal of Microbiology, 52(8), 701–716. 16917528 10.1139/w06-023

[ece311423-bib-0033] Osono, T. (2014). Diversity and ecology of endophytic and epiphytic fungi of tree leaves in Japan: A review. In V. C. Verma & A. C. Gange (Eds.), Advances in endophytic research (pp. 3–26). Springer.

[ece311423-bib-0034] Peng, R. , & Christian, K. (2005). Integrated pest management in mango orchards in the Northern Territory Australia, using the weaver ant, *Oecophylla smaragdina*, (Hymenoptera: Formicidae) as a key element. International Journal of Pest Management, 51(2), 149–155.

[ece311423-bib-0035] Peng, R. , & Christian, K. (2013). Do weaver ant (Hymenoptera: Formicidae) marks affect mango internal quality and storage life? Journal of Economic Entomology, 106(1), 299–304. 23448044 10.1603/ec12162

[ece311423-bib-0036] Põlme, S. , Abarenkov, K. , Henrik Nilsson, R. , Lindahl, B. D. , Clemmensen, K. E. , Kauserud, H. , Nguyen, N. , Kjøller, R. , Bates, S. T. , Baldrian, P. , Frøslev, T. G. , Adojaan, K. , Vizzini, A. , Suija, A. , Pfister, D. , Baral, H. O. , Järv, H. , Madrid, H. , Nordén, J. , … Tedersoo, L. (2020). FungalTraits: A user‐friendly traits database of fungi and fungus‐like stramenopiles. Fungal Diversity, 105(1), 1–16.

[ece311423-bib-0037] Prior, R. , Feige, A. , & Begerow, D. (2017). Antagonistic activity of the phyllosphere fungal community. Sydowia an International Journal of Mycology, 69, 183–198.

[ece311423-bib-0039] Qian, X. , Duan, T. , Sun, X. , Zheng, Y. , Wang, Y. , Hu, M. , Yao, H. , Ji, N. , Lv, P. , Chen, L. , Shi, M. , Guo, L. , & Zhang, D. (2018). Host genotype strongly influences phyllosphere fungal communities associated with *Mussaenda pubescens* var. alba (Rubiaceae). Fungal Ecology, 36, 141–151.

[ece311423-bib-0040] Qian, X. , Li, S. , Wu, B. , Wang, Y. , Ji, N. , Yao, H. , Cai, H. , Shi, M. , & Zhang, D. (2020). Mainland and Island populations of *Mussaenda kwangtungensis* differ in their phyllosphere fungal community composition and network structure. Scientific Reports, 10(1), 952. 31969602 10.1038/s41598-020-57622-6 PMC6976661

[ece311423-bib-0041] R Core Team . (2023). R: A language and environment for statistical computing. R Foundation for Statistical Computing.

[ece311423-bib-0042] Roux, O. , Céréghino, R. , Solano, P. J. , & Dejean, A. (2011). Caterpillars and fungal pathogens: Two co‐occurring parasites of an ant‐plant mutualism. PLoS One, 6(5), e20538. 21655182 10.1371/journal.pone.0020538 PMC3105098

[ece311423-bib-0043] Sapkota, R. , Knorr, K. , Jørgensen, L. N. , O'Hanlon, K. A. , & Nicolaisen, M. (2015). Host genotype is an important determinant of the cereal Phyllosphere mycobiome. New Phytologist, 207(4), 1134–1144. 25898906 10.1111/nph.13418

[ece311423-bib-0044] Stone, B. W. G. , Weingarten, E. A. , & Jackson, C. R. (2018). The role of the phyllosphere microbiome in plant health and function. In J. A. Roberts (Ed.), Annual plant reviews online (pp. 533–556). Wiley.

[ece311423-bib-0045] Tanabe, A. S. , & Toju, H. (2013). Two new computational methods for universal DNA barcoding: A benchmark using barcode sequences of bacteria, archaea, animals, fungi, and land plants. PLoS One, 8, e76910. 24204702 10.1371/journal.pone.0076910 PMC3799923

[ece311423-bib-0046] Thornham, D. G. , Smith, J. M. , Ulmar Grafe, T. , & Federle, W. (2012). Setting the trap: Cleaning behaviour of *Camponotus schmitzi* ants increases long‐term capture efficiency of their pitcher plant host, *Nepenthes bicalcarata* . Functional Ecology, 26(1), 11–19.

[ece311423-bib-0047] Toju, H. , Tanabe, A. S. , Yamamoto, S. , & Sato, H. (2012). High‐coverage ITS primers for the DNA‐based identification of ascomycetes and basidiomycetes in environmental samples. PLoS One, 7(7), e40863. 22808280 10.1371/journal.pone.0040863 PMC3395698

[ece311423-bib-0048] Unterseher, M. , Peršoh, D. , & Schnittler, M. (2013). Leaf‐inhabiting endophytic fungi of European beech (*Fagus sylvatica L*.) co‐occur in leaf litter but are rare on decaying wood of the same host. Fungal Diversity, 60(1), 43–54.

[ece311423-bib-0049] Vacher, C. , Cordier, T. , & Vallance, J. (2016). The phyllosphere: Microbial jungle at the plant–climate interface. Annual Review of Ecology, Evolution, and Systematics, 47(1), 1–24.

[ece311423-bib-0050] Voříšková, J. , & Baldrian, P. (2013). Fungal community on decomposing leaf litter undergoes rapid successional changes. The ISME Journal, 7(3), 477–486. 23051693 10.1038/ismej.2012.116 PMC3578564

[ece311423-bib-0051] Yamawo, A. , Katayama, N. , Suzuki, N. , & Hada, Y. (2012). Plasticity in the expression of direct and indirect defence traits of young plants of *Mallotus japonicus* in relation to soil nutritional conditions. Plant Ecology, 213(1), 127–132.

[ece311423-bib-0052] Yamawo, A. , Suzuki, N. , Tagawa, J. , & Hada, Y. (2012). Leaf ageing promotes the shift in defence tactics in *Mallotus japonicus* from direct to indirect defence: *Defence‐tactics shift in Mallotus* . Journal of Ecology, 100(3), 802–809.

[ece311423-bib-0053] Yamawo, A. , Tagawa, J. , Hada, Y. , & Suzuki, N. (2014). Different combinations of multiple defence traits in an extrafloral nectary‐bearing plant growing under various habitat conditions. Journal of Ecology, 102(1), 238–247.

[ece311423-bib-0054] Yao, H. , Sun, X. , He, C. , Maitra, P. , Li, X. C. , & Guo, L. D. (2019). Phyllosphere epiphytic and endophytic fungal community and network structures differ in a tropical mangrove ecosystem. Microbiome, 7(1), 57. 30967154 10.1186/s40168-019-0671-0 PMC6456958

