# Supplementary material for: Foraging ants affect community composition and diversity of phyllosphere fungi on a myrmecophilous plants, Mallotus japonicus
Source: Ecol Evol. 2024 May 15;14(5):e11423. doi: 10.1002/ece3.11423 (PMC11094773; doi:10.1002/ece3.11423)
Supplement: Supplementary file 2 — Appendix S2. [file ECE3-14-e11423-s001.pdf]

Appendix 2

Effect of experimental manipulation on ant species richness and composition on target leaves

We performed GLMM analysis and PERMANOVA to test if species richness and species composition of ants, respectively, varied after starting the field experiment. We did not use data of ant-exclusion treatment for these analyses because there was almost no ant on the target leaves after resin application.

Ant species richness

GLMM with Poisson distribution was performed with *glmer* function in *lme4* package. Species richness of each observation was set as a response variable. Treatment (two levels: resin-applied control and control), manipulation (two levels: before and after) and their interaction were set as explanatory variables. Although we did not conduct any manipulation on control plants, the observation at the first day were assigned to "before" manipulation. Plot and plant individuals were set as random factors. The best model was selected based on AIC and then significance of the explanatory variables in the best model was assessed with likelihood ratio test.

In control and resin-applied control, distributions of ant species richness were similar between before and after the manipulation (Appendix Fig.1). Both factors, treatment and manipulation, did not significantly affect ant species richness. The best model was model no.3 which has "treatment" as explanatory variable (Appendix Table.1). The effect of treatment in model no.3 was not significant (likelihood ratio test: $\chi^2 = 2.050$, $p = 0.152$).

Appendix Fig.1 Histograms of ant species richness between before and after the manipulation in the three treatments.

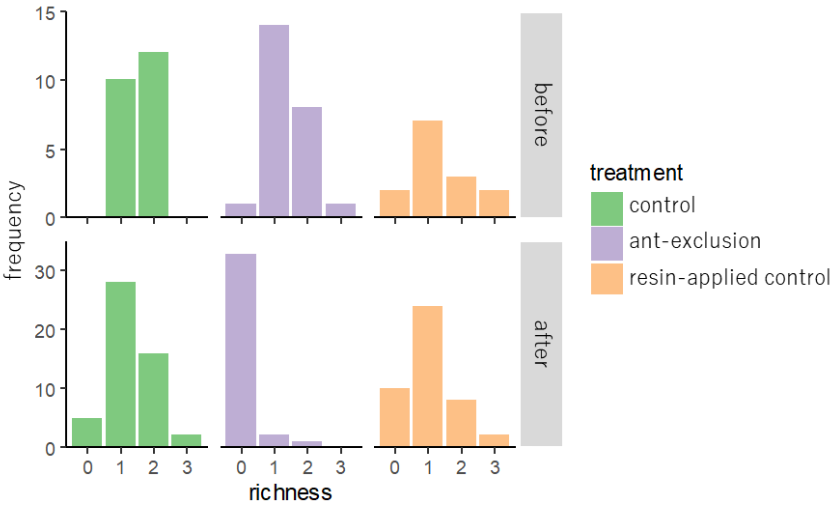

Appendix 2

Appendix Table.1 Fixed effects terms and AIC of the models for GLMM

model no.	fixed effect terms	AIC
1	treatment + manipulation + treatment:manipulation	634.1
2	treatment + manipulation	632.2
3	treatment	630.2
4	manipulation	632.3
5	1	630.3

Ant species composition

Two distance matrices of the ant community were constructed using Bray–Curtis dissimilarity and Jaccard dissimilarity. We performed a PERMANOVA with three explanatory variables, treatment (two levels: resin-applied control and control), manipulation (two levels: before and after) and their interactions. We set the plot as a block so that sample units were permuted within a block and ran PERMANOVA with 9999 permutations with the *adonis2* function. All functions used for the fungal community analyses described above were performed using *vegan* package.

The results showed treatment and manipulation did not affect ant species composition in analyses with both Bray–Curtis dissimilarity and Jaccard dissimilarity (Appendix Table.2).

Appendix Table.2 Results of PERMANOVA for ant species composition

		Df	Sum of Sqs	R^2	F	Pr (>F)
Bray–Curtis	treatment	1	0.711	0.015	1.746	0.425
	manipulation	1	0.586	0.013	1.438	0.156
	treatment:manipulation	1	0.684	0.015	1.681	0.081
	Residual	110	44.8	0.958		
Jaccard	treatment	1	0.638	0.014	1.611	0.573
	manipulation	1	0.596	0.013	1.505	0.139
	treatment:manipulation	1	0.6	0.013	1.515	0.132
	Residual	110	43.56	0.960		
